# End-of-life care in a psychiatric hospital

**DOI:** 10.1192/pb.bp.114.049833

**Published:** 2016-06

**Authors:** Lauren Z. Waterman, David Denton, Ollie Minton

**Affiliations:** 1 Sussex Partnership NHS Trust; 2 St George's University Hospitals NHS Foundation Trust

## Abstract

Since the Liverpool Care Pathway has been withdrawn in the UK, clinicians supporting the palliative needs of patients have faced further challenges, particularly for patients with dementia who are unable to go to a hospice owing to challenging behaviours. It is becoming more important for different services to provide long-term palliative care for patients with dementia. Mental health trusts should construct end-of-life care policies and train staff members accordingly. Through collaborative working, dying patients may be kept where they are best suited. We present the case study of a patient who received end-of-life care at a psychiatric hospital in the UK. We aim to demonstrate how effective end-of-life care might be provided in a psychiatric hospital, in accordance with recent new palliative care guidelines, and highlight potential barriers.

End-of-life care is defined as care that helps those with advanced, progressive, incurable illness to live as well as possible until they die, by focusing on their palliative care needs during their last phase of life.^1^ The end of the Liverpool Care Pathway, whose use has been withdrawn in the UK since July 2014,^2^ has brought challenges to clinicians supporting patients who are in need of palliative and end-of-life care, particularly for patients with dementia who are unable to go to a hospice owing to their challenging behaviours. It is essential that this problem be addressed. Dementia is now recognised as one of the world's most pressing public health issues,^3^ with 35.6 million people living with dementia in 2010 (and the number predicted to double by 2050),^4^ and increasing research into living with dementia is showing long periods of troubling symptoms in these patients.^5–7^ The Care Quality Commission is currently focusing on inequalities experienced by people in end-of-life care, and has identified people with dementia and other mental health needs as a particular priority group.^2^

At a psychiatric hospital in England, we found that patients with dementia with life-threatening, comorbid conditions and communication problems have particular difficulties in expressing their wishes and receiving optimal end-of-life care. There may also be barriers to keeping them in their place of preference and/or the place where they are at their most comfortable. We present a case study to illustrate these particular difficulties and suggest potential solutions.

## Case study

Mrs S was a 55-year-old patient on the dementia ward at a psychiatric hospital. For the majority of her life, Mrs S was high in functioning and had no history of mental illness. In 2012, she was diagnosed with amyotrophic lateral sclerosis (motor neurone disease), which predominantly affected the muscles responsible for speech and swallow. As her disease progressed she developed speech difficulties and her swallow was affected, therefore she was fed via percutaneous endoscopic gastrostomy (PEG) tube from 2013. She initially communicated in writing and via a communication book; however, her cognition declined and she developed expressive and receptive aphasias. She began to exhibit erratic behaviour and was diagnosed with frontotemporal dementia by a neurologist in June 2013. Her medication consisted of riluzole, dietary supplements and antisecretory medications.

### Admission to a psychiatric hospital

Mrs S's admission to the psychiatric hospital came as a result of self-harm, self-neglect and vulnerability. She had begun pulling out her PEG feeding tube, eating and drinking orally (which posed a serious health risk), going out for walks late at night and refusing the assistance of her carers.

She declined admission to hospital, but for her safety was admitted under Section 2 of the Mental Health Act 1983. She had made an advance decision in August 2013 stating that she wished to die at home and did not want to go into residential care; that she would want to receive antibiotics in hospital for chest infections, but that she did not wish to be ventilated or resuscitated. However, when it came to making decisions about placement and when to cease life-prolonging treatment, her family felt that she did not have capacity at the time of making the directive. It was made when she had already developed aphasia, and although she was able to nod or shake her head to questions, there was inconsistency regarding her responses; this was also mentioned in the report of a speech and language therapist in September that year. The advance decision was therefore deemed not to be valid by the multidisciplinary team, in accordance with the Mental Capacity Act 2005.

### The patient's time on the dementia ward

During her time on the ward, Mrs S displayed emotional lability, exhibited possible suicidal intent, and was subsequently started on the antidepressant citalopram. She was switched to Section 3 of the Mental Health Act and then discharged from that section in November as she was agreeable to staying in hospital and receiving treatment.

As time progressed, she became more distressed owing to excess respiratory secretions. In September 2013 she had been admitted to the general medical hospital for a month for a lower respiratory tract infection; during this time, the local hospice and palliative care team were involved and became familiar with her. Therefore, we sought medication and management advice from the nurses and palliative medicine consultant from the hospice, who came to review Mrs S on a couple of occasions. In 2014 she was readmitted to the county hospital due to bronchiectasis.

### End-of-life care planning

After Mrs S's subsequent discharge from the county hospital, a review meeting was organised involving her family and ward team. Her family had found this last admission distressing, particularly since Mrs S's secretions had been suctioned via nasogastric tube in the accident and emergency resuscitation bay when she was biting on the suction tube during oral attempts. Her family did not wish her to be admitted to hospital again or to have further life-prolonging treatment, although there was some confusion about the role that oral antibiotics might play, given her advance directive stating her wish to receive them. Her family wished her to die peacefully at the psychiatric hospital, with which she had familiarised herself over the months. She could not be accepted at a hospice while she was still mobile due to her high risks.

A further multidisciplinary meeting was organised, this time with the palliative medicine consultant from the local hospice and nurses from Hospice at Home present, as well as Mrs S's family and ward staff. Since the patient's advance decision had been deemed invalid, a best interests decision was made, incorporating the views of her family and healthcare professionals, in accordance with the Mental Capacity Act 2005: this was to stop life-prolonging treatment. Specific plans were defined clearly in a comprehensive care plan, which was finalised by the ward nursing staff. Examples of these plans were: to suction secretions and give oxygen only if the patient is accepting of these; not to sedate for the purpose of suctioning, but if the patient were to be sedated for other reasons then suction could be attempted; family visits to be allowed whenever wanted; end-of-life palliative medications to be pre-emptively prescribed and ordered; physical observations to stop; not to be transferred to a medical hospital. Transfer to hospice would be considered if the patient were to become bed-bound and if staff at the psychiatric hospital were unable to care effectively for her needs. The nurses from the Hospice at Home team agreed to support the nursing staff with providing effective end-of-life care to Mrs S, and to come in to assess her symptomatically and administer medications via a syringe driver. We emphasised that since the ward staff were not sufficiently trained or experienced in this type of care, the external nurses would need to treat the situation in the same way as if Mrs S was dying at home. They were happy to do this. Fortunately, we were also able to bring in a registered general nurse to provide care during this time. We invited a chaplain to the ward to provide spiritual support to the patient and her family. During March 2014, Mrs S began to spend more time in bed, although there were still periods where she was active and would pace down the corridor. She passed away in March 2014.

The patient's family were grateful that we had allowed her to die in a familiar environment, as this familiarity was crucial in supporting her psychological well-being. Following her death, many staff members on the ward were distressed by her passing, having spent many months working closely with her and not being used to patients dying on the ward. A debriefing session for staff was organised so that they could discuss their emotions in an appropriate environment.

## Discussion

This case highlights how effective end-of-life care might be provided in a psychiatric hospital, to allow a patient to die being as comfortable as possible in an environment they are familiar with and to achieve continuity of care. In this setting, staff may not be familiar with how to provide optimal end-of-life care, but it can be provided with sufficient palliative care team input and good multidisciplinary working. Continuity of care is one of the key recommendations for achieving optimal palliative care in people with dementia that was made by the European Association of Palliative Care,^8^ as the result of a five-round Delphi study involving 23 countries. The European Commission also declared in their summary report from the European Union summit on chronic diseases (April 2014) that, for an ageing society, ‘more investment and innovation are needed to redesign and adapt care systems, especially by fostering better integration of services and ensuring the continuity of care’ (p. 2).^9^

This case is presented in light of new national guidance for end-of-life care provision. The Liverpool Care Pathway had been criticised for facilitating poor communication and decision-making, with ‘paternalistic failure’ seen as a key feature in its demise.^10^ Now, guidance published by NHS England^11^ and yet more recent guidance from the Leadership Alliance for the Care of Dying People^2^ remind clinicians that good communication is key and that it should be ensured that patients and relatives are more central to decision-making. The Alliance also states that an individualised care plan for each patient must be coordinated by the senior clinicians involved in their care – for any patients who are dying in a psychiatric hospital, this means the consultant psychiatrists responsible for that patient.^2^

The Alliance has declared five priorities for providing good end-of-life care, which we were able to address as follows.

- Priority 1: ‘This possibility [that a person may die within the next few days or hours] is recognised and communicated clearly, decisions made and actions taken in accordance with the person's needs and wishes, and these are regularly reviewed and decisions revised accordingly’. We benefited from the expertise of the community palliative care consultant and nurses in diagnosing when the patient was imminently dying, and we consequently communicated to her family that she was dying. We regularly reviewed the patient's care plan via multiple multidisciplinary meetings.
- Priority 2: ‘Sensitive communication takes place between staff and the dying person, and those identified as important to them’. Good communication with family members was key in Mrs S's case, and allowed us to produce a thorough care plan that was based on the patient's best interests. As we had already got to know the patient's family well during her time on the ward, this made these conversations easier, as her family felt able to be open with us about their concerns. Again, the local palliative care team helped us to achieve this goal by answering some of the more technical questions about dying that the family asked.
- Priority 3: ‘The dying person, and those identified as important to them, are involved in decisions about treatment and care to the extent that the dying person wants’. We faced difficulties in addressing this priority, as the patient had already lost the capacity to make decisions about her treatment and the advance decision was deemed invalid. All attempts were made by the medical team and speech and language specialist to enable the patient to communicate her wishes, but these were not successful. This issue was discussed with her close relatives. The relatives were included in multidisciplinary meetings and we ensured that they had ample opportunity to voice their opinions and concerns to all the professionals involved in the patient's care.
- Priority 4: ‘The needs of families and others identified as important to the dying person are actively explored, respected and met as far as possible’. The views of the patient's family were taken into account, such as that they were keen for her to remain on the dementia ward. They were very grateful to have their views listened to and considered. They were involved in best interests meetings about the patient and their opinions were actively sought out. We ensured their questions about the dying process could be answered comprehensively by inviting the palliative care consultant to a meeting with them, and they were offered the support of a chaplain.
- Priority 5: ‘An individual plan of care, which includes food and drink, symptom control and psychological, social and spiritual support, is agreed, co-ordinated and delivered with compassion’. Following multidisciplinary meetings, a comprehensive personalised care plan was constructed and reviewed at a later meeting. The Hospice at Home team provided assistance with symptom control, by helping the ward nursing staff to set up a syringe driver. The patient was able to communicate non-verbally when she was hungry, therefore food and drink was reserved for when she requested it. Intravenous fluids and excessive fluids via her PEG were avoided, as this would exacerbate her upper airway secretions. Psychosocial support was provided by keeping the patient in a place where she was familiar to and comfortable with the staff. She was permitted the freedom to pace the hallways freely as she wished, supervised by nursing staff. Spiritual support was offered via a chaplain.

The Hospice at Home team's input was essential to the care of Mrs S. It is helpful for wards to bring in a registered general nurse temporarily if they do not already have one. Additionally, debriefing for staff after Mrs S's death was considered essential, since the staff, having become close with her over many months, were affected by her death.

A major issue that we encountered was that Mrs S's advance decision was not valid. This is because she had made the decision at a time where it was deemed she lacked capacity to accept or refuse medical treatment. Her case highlights the importance of early advance care planning: it should be encouraged at the time of diagnosis of dementia or any other terminal illness, rather than at a time where capacity to make important decisions may have already been lost.^12^ This has been emphasised in guidelines from the National Council of Palliative Care,^12^ and two international Delphi studies have shown very high consensus between specialists that planning of future care should begin as soon as dementia has been diagnosed.^8,13^ Key principles of advanced care planning have been published by the University of Nottingham,^14^ which stress the importance of introducing discussions sensitively, assessing the capacity of the individual to understand and discuss the options for future treatment, thorough record-keeping and regular review of decisions. Feeding by tube or drip is considered a form of medical treatment,^15^ therefore decisions regarding the instigation or continuation of clinically assisted nutrition should also be discussed during advanced care planning, particularly in a case such as this where the patient was likely to lose her ability to swallow. A barrier to early advanced care planning is that individuals with dementia, their families and healthcare professionals may not always recognise dementia as a terminal illness. In fact, a recent Australian study published in the *Journal of Palliative Care* showed that around 50% of nursing home staff and 60% of family caregivers did not consider dementia to be a terminal illness.^16^ Therefore, it is vital to provide education about the illness trajectory of dementia as soon as possible after diagnosis, to facilitate early advanced care planning.

Another issue highlighted by this case was that it is important for mental health trusts that include in-patient wards to establish trust end-of-life care policies. In creating these, it is advantageous to learn from care homes and palliative care centres about end-of-life care for patients with dementia: this is particularly pressing since research into palliative care in dementia has been limited and mostly consensus-based.^17^ The nursing staff at the psychiatric hospital may have found it easier to provide care to Mrs S if they had more specific and targeted end-of-life care guidelines to refer to, targeted at providing care in a psychiatric hospital. There may also be a need to train staff on dementia wards in caring for dying patients. As all dementia services become more stretched with rates of dementia increasing (and people living longer with dementia),^3^ it is likely to become even more important for different services to provide long-term palliative care for these patients. The financial costs of providing end-of-life care in a psychiatric hospital are large, due to the need to keep the patient on close observation, long-term hospital stay, occupying the time of multiple staff members with multidisciplinary meetings, and employment of a full-time registered general nurse if one is not already employed. However, our case, along with the recent guidelines,^2^ highlights the need to be flexible with where patients die and discuss the options in the multidisciplinary setting with the family. Hospices cannot always cater for patients with dementia due to their behavioural difficulties and transfer out of a psychiatric hospital may not always be in the patient's best interests. Therefore, palliative care and dementia care teams need to have flexibility in adapting to the changing needs of people with dementia, and to thus explore and evaluate new approaches to providing end-of-life care. There also needs to be further collaboration between palliative care and dementia specialists, and between NHS England, third-sector organisations, medical Royal Colleges and the Department of Health to meet this challenge. Through collaborative working, it may be possible to keep dying patients in the place where they are best suited.

## References

[1] National Council for Palliative Care Commissioning End of Life Care: Act and Early. Initial Actions for New Commissioners. NCPC, 2011.

[2] Leadership Alliance for the Care of Dying People Once Chance to Get it Right: Improving People's Experience of Care in the Last Few Days and Hours of Life. LACDP, 2014.

[3] World Health Organization Dementia: A Public Health Priority. WHO, 2012.

[4] Alzheimer's Disease International World Alzheimer Report 2010: The Global Economic Impact of Dementia. International Federation of Alzheimer's Disease and Related Disorders Societies, 2010.

[5] Meeussen K Van den Block L Echteld M Boffin N Bilsen J Van Casteren V Older people dying with dementia: a nationwide study. Int Psychogeriatr 2012; 24: 1581–91. 22647226 10.1017/S1041610212000865

[6] Van Uden N Van den Block L van der Steen JT Onwuteaka-Philipsen BD Vandervoort A Vander Stichele R Quality of dying of nursing home residents with dementia as judged by relatives. Int Psychogeriatr 2013; 25: 1697–707. 23735194 10.1017/S1041610213000756

[7] Vandervoort A Van den Block L van der Steen JT Volicer L Vander Stichele R Houttekier D Nursing home residents dying with dementia in Flanders, Belgium: A nationwide post-mortem study on clinical characteristics and quality of dying. J Am Med Dir Assoc 2013; 14: 485–92. 23523319 10.1016/j.jamda.2013.01.016

[8] van der Steen J Radbruch L Hertogh C Boer M Hughes J Larkin P White paper defining optimal palliative care in older people with dementia: a Delphi study and recommendations from the European Association for Palliative Care. Palliat Med 2014; 28: 197–209. 23828874 10.1177/0269216313493685

[9] European Commission The 2014 EU Summit on Chronic Diseases: Conference Conclusions. European Commission, 2014.

[10] Neuberger J Guthrie C Aaronovitch D More Care Less Pathway: A Review of the Liverpool Care Pathway. Department of Health, 2013.

[11] NHS England Guidance for Doctors and Nurses Caring for People in the Last Days of Life. National Council for Palliative Care, 2013.

[12] National Council for Palliative Care Capacity, Care Planning and Advance Care Planning in Life Limiting Illness. NCPC, 2011.

[13] Annear M Toye C McInerney F Eccleston C Tranter B Elliott KE What should we know about dementia in the 21st century? A Delphi consensus study. BMC Geriatrics 2015; 15: 5. 25656075 10.1186/s12877-015-0008-1 PMC4326452

[14] Henry C Seymour J Ryder S Advance Care Planning: A Guide for Health and Social Care Staff. University of Nottingham, 2007.

[15] General Medical Council Treatment and Care Towards the End of Life: Good Practice in Decision Making. GMC, 2010.

[16] Robinson A Eccleston C Annear M Elliott KE Andrews S Stirling C Who knows, who cares? Dementia knowledge among nurses, care workers, and family members of people living with dementia. J Palliat Care 2014; 30: 158–65. 25265739

[17] van der Steen JT Dying with dementia: what we know after more than a decade of research. J Alzheimers Dis 2010; 22: 37–55. 20847433 10.3233/JAD-2010-100744

